# H-aggregate analysis of P3HT thin films-Capability and limitation of photoluminescence and UV/Vis spectroscopy

**DOI:** 10.1038/srep32434

**Published:** 2016-09-01

**Authors:** Philipp Ehrenreich, Susanne T. Birkhold, Eugen Zimmermann, Hao Hu, Kwang-Dae Kim, Jonas Weickert, Thomas Pfadler, Lukas Schmidt-Mende

**Affiliations:** 1 Department of Physics, University of Konstanz, POB 680, 78457 Konstanz, Germany

## Abstract

Polymer morphology and aggregation play an essential role for efficient charge carrier transport and charge separation in polymer-based electronic devices. It is a common method to apply the H-aggregate model to UV/Vis or photoluminescence spectra in order to analyze polymer aggregation. In this work we present strategies to obtain reliable and conclusive information on polymer aggregation and morphology based on the application of an H-aggregate analysis on UV/Vis and photoluminescence spectra. We demonstrate, with P3HT as model system, that thickness dependent reflection behavior can lead to misinterpretation of UV/Vis spectra within the H-aggregate model. Values for the exciton bandwidth can deviate by a factor of two for polymer thicknesses below 150 nm. In contrast, photoluminescence spectra are found to be a reliable basis for characterization of polymer aggregation due to their weaker dependence on the wavelength dependent refractive index of the polymer. We demonstrate this by studying the influence of surface characteristics on polymer aggregation for spin-coated thin-films that are commonly used in organic and hybrid solar cells.

Polymer-based electronics have attracted increasing research interest within the last decades. Not only its low-cost potential, but also the emerging field of applications like flexible electronic displays strongly influence this development^1^,^2^. Though some applications have been established successfully, a broad usage still requires a deeper understanding of the influence of polymer arrangement on the electronic properties of the device. Improving charge carrier transport towards the electrodes is a major requirement for all polymer-based devices such as transistors, organic light-emitting diodes or organic solar cells. Charge transfer in conjugated polymers takes place either along the polymer backbone (intrachain transport), or across π-orbital coupled neighboring polymer chains (interchain transport). Whereas intrachain transport is mainly affected by the alignment of the polymer backbone, interchain transport critically depends on the order in π-stacking direction. Charge transfer in either direction is disrupted by disorder^3^, which can break the delocalization of wavefunctions due to non- or weakly overlapping atomic orbitals. Most commonly, intrachain transport along the polymer backbone demonstrates a higher mobility compared to interchain transport in π-π stacking direction, with a difference of about two orders of magnitude in case of poly(3-hexylthiophene)^4^,^5^,^6^. For this reason it is necessary to characterize polymer systems with regard to their complex morphology behavior. One very promising and widely used technique^7^,^8^,^9^,^10^ for such a characterization is offered by Spano and co-workers^9^,^10^,^11^,^12^,^13^,^14^, who have developed the HJ-aggregate model. It allows to investigate polymer aggregates with respect to their structural order with commonly used experiments like UV/Vis and photoluminescence (PL) measurements. With the help of those measurements it is possible to gain insights in the relative transition dipole orientations within the polymer film, which are governed by the alignment of the polymer chains. Spano and Clark *et al.*^11^,^13^ have implemented Equation (1) in order to determine the exciton bandwidth *W*, by comparing the amplitude ratio of the vibronic 0-0 transition (*A*_*0-0*_) to the 0-1 transition (*A*_*0-1*_). Both transitions are separated by the energy (*E*_*P*_ = 0.17 eV) of a C = C stretching mode:




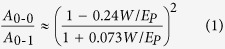




It is established that the exciton bandwidth *W* is given by the nearest-neighbor interchain Coulombic coupling^10^. As recently pointed out by Spano and co-workers, a competition between interchain (H-aggregates) and intrachain (J-aggregates) interaction exists in aggregated polymer films^10^,^15^. In comparison to small molecules that can be directly assigned to H-aggregates, polymer films are inherently two-dimensional excitonic systems with both inter- and intrachain interactions. Depending on crystal size, molecular weight or polymer order, either of them can be dominant^10^,^15^. In the limit of pure H-aggregates, the 0-0 transition is dipole forbidden, expressed through a large exciton bandwidth. In J-aggregates, however, this transition is super-radiative. Also disorder, which is significant in polymer films, can break the symmetry and enhance contributions of the 0-0 transition in an H-aggregate^10^. For films of similar interchain order, W can be related to the conjugation length, i.e., an increase in intrachain order expressed through a decrease in W. Although equation (1) has been introduced in the limit of H-aggregates, it has been shown to be valid also for increasing contributions of J-aggregates for spin-cast polymer films^15^.

From an experimental point of view, however, the H-aggregate model cannot always be directly applied to UV/Vis and photoluminescence spectra, as we will show in this work. Here, we investigate films with different thicknesses of the model polymer poly(3-hexylthiophene) (P3HT) spin-casted on substrates with varying roughness and chemical composition of the substrate surface. We compare experimental UV/Vis and PL spectra to simulated spectra based on a transfer matrix algorithm. We discuss the potential and challenges to experimentally obtain a quantitative analysis of UV/Vis and PL spectra within the H-aggregate framework. For this purpose, it is necessary to investigate the influence of the dispersive refractive index by means of various film thicknesses.

## Results and Discussion

**Figure 1:**
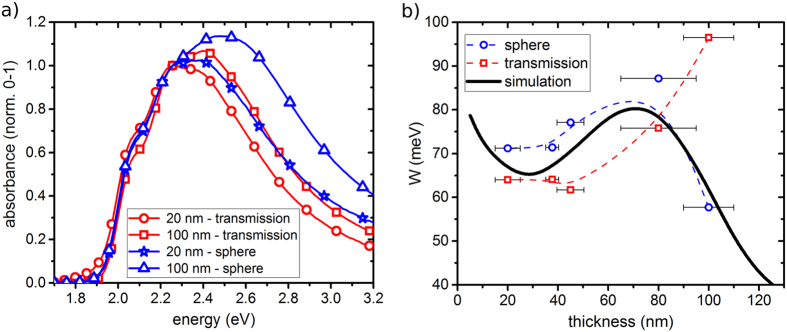
(**a**) UV/Vis measurements of two different P3HT film thicknesses on borosilicate glass, measured in transmission (red, circle: 20 nm, square: 100 nm) and inside an integrating sphere (blue, stars: 20 nm; triangles: 100 nm). Spectra are normalized to the 0-1 transition at 2.25 eV. (**b**) Exciton bandwidth W, as a function of film thickness determined from absorbance measurements in transmission (red squares) and in an integrating sphere (blue circles)–dashed lines represent a b-spline for qualitative illustration. The solid black line represents the exciton bandwidth W_calc_ determined from simulated absorbance spectra using the transfer matrix algorithm published by Burkhard *et al*.^24^

The thickness of a P3HT film is adjusted by spin-casting solutions of varying polymer concentration. This is a commonly used and an appropriate method^16^,^17^,^18^ for low concentrations, in which the intermolecular interaction in solution is minimal^19^. In general, the lower the concentration, the thinner the polymer film. Consequently, the relative contribution of the interfacial polymer configuration to the overall PL or UV/Vis spectrum can be revealed, i.e., it is possible to differentiate between bulk and surface effects. For solar cell application, the polymer alignment at an interface to an acceptor/donor material is very essential. Efficient charge separation as well as charge transport away from the interface require high polymer crystallinity and a face-on aggregate orientation to ensure charge delocalization at this donor-acceptor interface^20^,^21^,^22^. By changing the surface characteristics we are able to reveal a possible pathway to influence the polymer aggregation directly. In particular, the first monolayer energetics are very important to control the efficiency of charge transport^16^ and the energy of charge transfer states (CTSs), which affects geminate/non-geminate recombination^23^. In order to keep adhesive forces between different polymer solutions and a given type of substrate comparable, the polymer concentration is varied in a limited range from 5 to 20 mg/ml dissolved in chlorobenzene (CB). Figure 1(a) shows UV/Vis spectra of P3HT deposited on flat borosilicate glass substrates (R_RMS_ < 2 nm). UV/Vis spectra of a 100 nm (20 mg/ml) and a 20 nm (5 mg/ml) film are presented, that are either characterized by detecting the transmitted light (i.e., transmission) or by detecting both transmitted and reflected light (i.e., integrating sphere). In transmission, we observe a significant reduction in the 0-0 transition at 2.05 eV for the 100 nm polymer film. In addition, there is a distinct increase of the amorphous absorption shoulder (between 2.4–3.2 eV). Since there is no additional spectral blue shift, these results suggest stronger intramolecular interactions and an increased fraction of polymer aggregates in the thinner film. These observations change, when both films are measured inside an integrating sphere. Here, we additionally take reflected light into account and accurately obtain the absorbance. Similar to the measurement in transmission, the trend in the amorphous shoulder is very pronounced. In contrast, differences in the 0-0 transition are negligible. Consequently, reflection has a significant impact on the measured absorbance spectra and has to be taken into account for a quantitative analysis. In order to better visualize the impact of reflection on an H-aggregate analysis, the exciton bandwidth *W* is determined with Equation (1). In Fig. 1(b), we show corresponding values for both measurement modes depending on film thickness, which is determined with an AFM. A series of film thicknesses is tested to resolve a systematic behavior. Below 50 nm the exciton bandwidth *W*, determined from spectra measured in transmission (red squares), shows no dependence on film thickness. Above 50 nm, the exciton bandwidth *W* is increasing significantly, which is consistent with first principle observations in Fig. 1(a). Contrary to that, the evaluation of absorbance measurements in an integrating sphere result in a continuously increasing exciton bandwidth *W* for increasing film thicknesses up to 80 nm. With further increasing film thickness the value for the exciton bandwidth is decreasing again. We compare these results to simulated absorbance spectra of P3HT on glass using the transfer matrix algorithm published by Burkhard *et al.*^24^. Thereby, the light propagation is simulated for varying thicknesses of P3HT with a given dispersion relation of the extinction coefficient. Corresponding absorbance and reflection spectra are shown in Figure S1 in the supplementary information. The resulting exciton bandwidth *W* from the calculated spectra is shown in Fig. 1(b) as solid line. Importantly, also here the exciton bandwidth *W*_*calc*_ is not constant, although the morphology is assumed to be unchanged. For film thicknesses up to 80 nm, *W*_*calc*_ shows an oscillating behavior and decreases monotonically for thicker films. These results show that *W*_*calc*_ coincides well with the experimentally determined *W* using the integrating sphere measurement. This behavior is a direct consequence of a varying reflection behavior depending on the film thickness. The simulated reflection spectra presented in Figure S1 reveal a clear thickness dependence. As a result, absorbance spectra evoke the impression that the exciton bandwidth is dependent on film thickness, although *W* is a function of the electronic coupling between polymer chromophores only. Each time the incident light beam encounters a polymer/substrate or polymer/air interface, a portion of light is transmitted out of the film and measured as contribution to the overall reflection, while the remaining portion is reflected into the film again. The influence of reflection on absorbance measurements has also been reported by Gaudin *et al.*^25^. In order to determine possible changes in aggregation with UV/Vis measurements, it is therefore essential to keep the film thickness constant or to check for possible film thickness variations. Based on both experimental and theoretical observations, deviations in the exciton bandwidth by more than 100% can occur for thicknesses below 150 nm. Even a small variation in thickness from 80 to 100 nm can affect the exciton bandwidth by more than 25%, though the polymer aggregation has not changed at all. Furthermore, due to the spectrally dependent reflection, it can also be difficult to quantify the ratio between aggregated and non-aggregated polymer in a film by simply subtracting the absorption contribution of the amorphous polymer. Additional variations in reflection can occur, if the polymer morphology is changing, since the index of refraction strongly depends on the degree of polymer aggregation^26^. Therefore, it is also challenging to determine polymer aggregation in a blend with varying fraction of other materials using this technique. Not only the film thickness, but also the refractive index is supposed to change in that case and the contribution of reflection in both films is not comparable. The same argument holds for temperature dependent spectra, since not only the film thickness is decreasing with decreasing temperature but also the refractive index changes^27^. For this reason, a quantitative analysis based on UV/Vis spectra in terms of absolute film morphology and aggregation is highly complex. Since the portion of reflection changes also spectrally with film thickness, it is not possible to obtain any absolute information of aggregation and polymer morphology by UV/Vis measurements, even if reflection is taken into account. Our analysis shows, that reflection for any film thickness impacts absorbance spectra in such a way that transitions relevant for an H-aggregate analysis are always affected. As the spectral range, where amorphous polymer morphologies absorb, is even stronger affected by the impact of reflection, we conclude that absorbance measurements do not allow for any quantitative conclusion about the ratio of aggregates to non-aggregates. Although it is sophisticated to obtain reliable data, we suggest ellipsometry measurements instead, where the extinction coefficient is determined with respect to the film thickness and changes in the real part of the refractive index are simultaneously tracked. The extinction coefficient monitors directly the intrinsic exciton bandwidth and is therefore a quantitative measure for *W*.

Another possibility to characterize polymer aggregation within the H-aggregate model are PL measurements^10^,^15^. The main difference between UV/Vis and photoluminescence spectra lies in their origin, i.e., while the whole polymer matrix is absorbing (aggregated and non-aggregated domains) only energetically low lying aggregates are involved in emission processes after subsequent energy transfer to these domains. However, since PL is the inverse physical process to absorption, application of the above discussed H-aggregated model on emission spectra stays valid and similar conclusion can be drawn with regard to the nature of aggregates and its surrounding.

**Figure 2:**
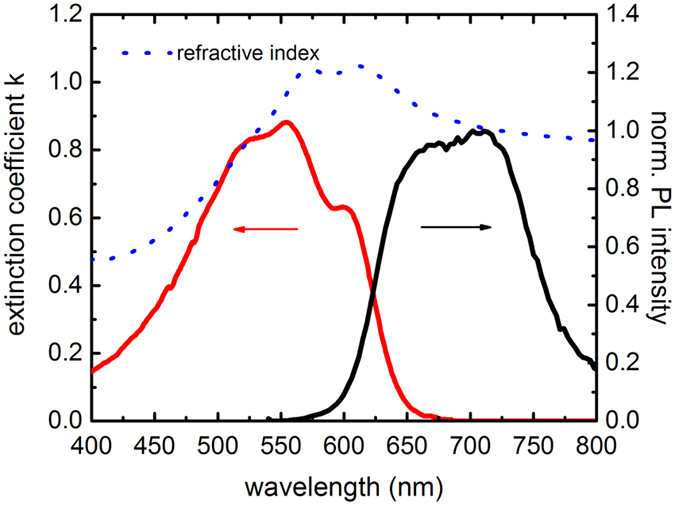


**Figure 3:**
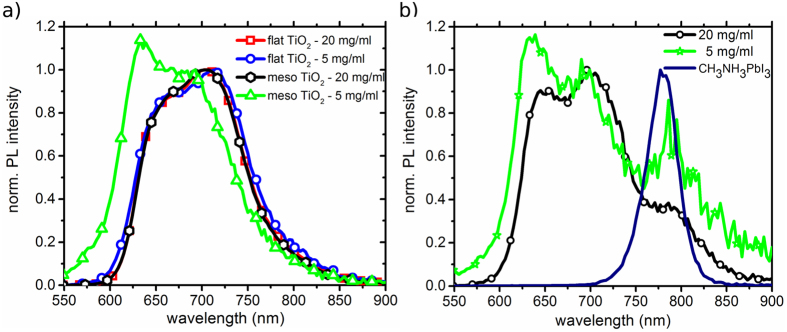
(**a**) Normalized PL spectra to the 0-1 transition of P3HT for two different film thicknesses using flat TiO_2_ (red square: 20 mg/ml, blue circles: 5 mg/ml) and mesoporous TiO_2_ (black hexagons: 20 mg/ml, green triangle: 5 mg/ml); (**b**) PL spectra of a thick (black circles: 20 mg/ml) and a thin (green stars: 5 mg/ml) P3HT layer on perovskite. The CH_3_NH_3_PbI_3_ perovskite PL is illustrated as a blue line.

In case of organic materials, PL spectra are less affected by dispersion compared to UV/Vis spectra, since the PL is aloof from a resonance in the dielectric function due to the Stokes-shift. This different dependence on the refractive index compared to absorbance spectra is illustrated for the example of P3HT in Fig. 2. As can be seen, the refractive index is increasing significantly in the spectral region where the polymer is absorbing, whereas this change is much smaller in the region of the photoluminescence. One has to keep in mind that this small change in refractive index has still an impact on the PL spectrum, as spectral variations in the refractive index also here influence the efficiency of light out-coupling of the PL at the polymer/air interface. However, in our measurements on P3HT this effect is only of minor importance, because the observed change in 0-0/0-1 amplitude ratio is affected by less than 5% in a detection angle regime which is not close to the limit of total reflection (for more details see supplementary information). Furthermore, PL spectra are insensitive to variations in film thickness, such that spectral changes can be directly related to changes in the polymer aggregation, which is not the case for UV/Vis measurements as explained above. We show this exemplarily in Fig. 3(a) by comparing varying thicknesses of spin-casted P3HT films on a v (film consisting of 20 nm nanoparticles sintered together to form a ~500 nm mesoporous film with root mean square surface roughness R_RMS_ = 20.1 nm) and a flat TiO_2_ surface (R_RMS_ = 1.62 nm).

**Figure 4:**
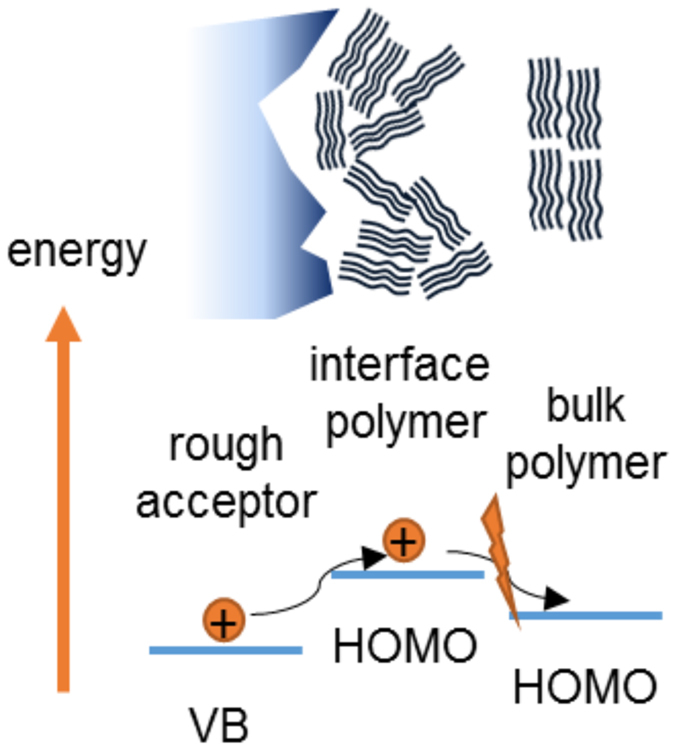


In the following the film thickness is assumed to directly correlate with the polymer concentration. An absolute thickness determination is experimentally challenging for bilayers featuring a rough interface, but also not necessary for a qualitative comparison of the evolution of polymer aggregation with film thickness for one specific substrate. Additionally, a comparison between different substrates is not possible due to potential differences in wetting and drying behavior, but rather the overall qualitative behavior for one type of substrate can be analyzed. In Fig. 3(a), PL spectra of P3HT films are presented using mesoporous and flat TiO_2_ substrates, which are coated with a thick (20 mg/ml) or a thin (5 mg/ml) P3HT layer. For flat substrates the difference in the spectra is marginal, suggesting only little influences on the polymer aggregation. Similarly, a thick P3HT film on mesoporous TiO_2_ does not show significant differences compared to the flat TiO_2_ case. A thin P3HT film on mesoporous TiO_2_, however, exhibits a substantial increase in the 0-0 transition, the peaks broaden and the spectrum shifts to the blue. Hence, we attribute this behavior to both a decrease in interchain interaction and an increase of the amorphous polymer phase towards a rougher substrate surface, whereas the bulk polymer morphology is less affected by these interfacial influences. In Fig. 3(b), the generalization of this observation in case of rough surfaces is shown for P3HT films cast on a CH_3_NH_3_PbI_3_ perovskite film (R_RMS_ > 20 nm), a device configuration often found in perovskite solar cells^28^,^29^. Besides the polymer luminescence, there is some residual contribution of perovskite PL that overlaps the spectrum of P3HT at 780 nm. The spectral blue shift and the increase of the 0-0 transition relative to the 0-1 transition indicate a more amorphous polymer matrix close to the interface with less intermolecular interaction. As illustrated in Fig. 4, this vertical phase separation can be a limiting factor for efficient charge transport since the HOMO level is energetically lower for a more crystalline polymer morphology, like in the bulk^30^,^31^. It can result in a loss channel since the hole transport is suppressed and charge generation is getting less efficient due to a loss of wavefunction delocalization at the interface as discussed by Herrmann *et al.*^21^. In particular for perovskite based solar cells, which exhibit large grain sizes and a significant surface roughness, such hindered charge transport in the hole conducting material away from the interface can result in increased charge carrier recombination.

**Figure 5:**
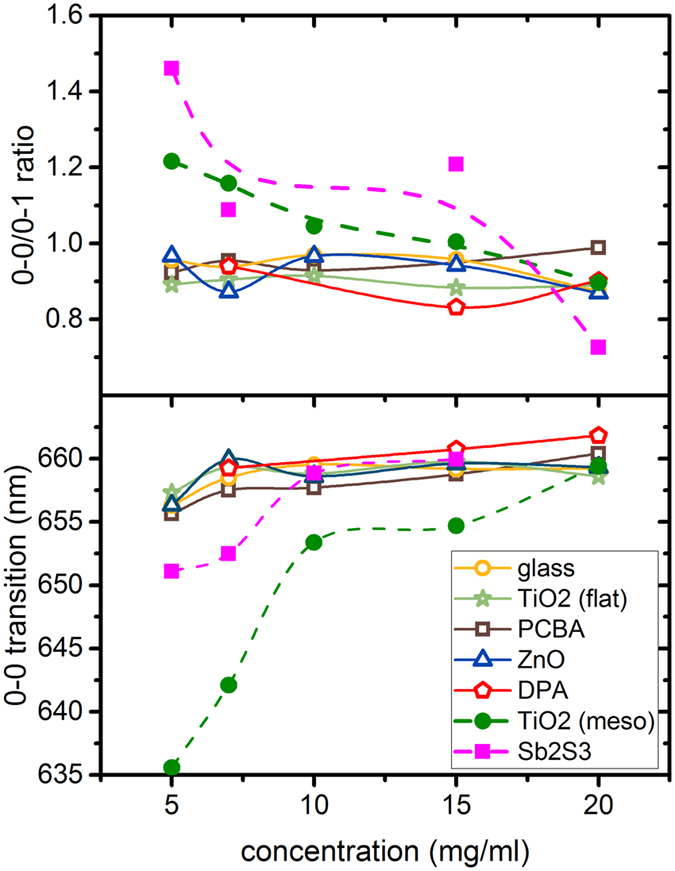
Comparison of P3HT photoluminescence characteristics on films of varying thickness on different substrates. Top: 0-0/0-1 transition amplitude ratio, bottom: spectral position of the 0-0 transition; Spectra have been fitted with 3 Gaussians of equal width. (dashed lines serve as guide to the eye).

To further evaluate the applicability of PL analysis within the H-aggregate model, we investigate the influence of different surface characteristics on polymer aggregation, including the impact of hydrophobic and hydrophilic surfaces as well as the possibility of a chelation process. Different common films that are often used in hybrid solar cells are used as substrate base for our P3HT films. Figure 5 shows the amplitude ratio of the 0-0 to 0-1 transition and the spectral position of the 0-0 transition as a function of polymer concentration. For Sb_2_S_3,_ we see a slight red shift of the 0-0 transition and a relatively high 0-0/0-1 amplitude ratio that is even larger than for the mesoporous TiO_2_. Although the film roughness is relatively small (R_RMS_ = 3.1 nm) compared to mesoporous TiO_2_, we observe a similar behavior with increasing aggregation away from the interface. However, the red shift is much weaker and the 0-0/0-1 transition ratio shows a maximum value for the thinnest film. This can be explained by an increase in the conjugation length, i.e., stronger J-aggregate interaction towards the interface. Since in J-aggregates the 0-0 transition is super-radiative^10^, we attribute these observations to the chelation interaction as described by Im *et al.*^32^, where thiophene moieties interact with Sb atoms at a Sb_2_S_3_ surface. Additionally, we investigate the polymer aggregation on flat substrates (R_RMS_ < 2 nm) of ZnO, TiO_2_, 1-decylphosphonic acid (DPA) modified TiO_2_ and [6, 6]-Phenyl C_61_ butyric acid (PCBA) modified TiO_2_. The 0-0/0-1 transition ratio stays approximately constant with varying film thickness of the polymer for all these surfaces and there is no significant impact by any modifier detectable. Neither DPA, making the surface more hydrophobic, nor PCBA change this ratio compared to bare TiO_2_. These results are in good agreement with findings of Chabinyc *et al.*^33^, who have investigated the influence of an octyltrichlorosilane monolayer on SiO_2_ on the polymer morphology. It is concluded that adhesion plays only a minor role for the size of the aggregates, hence the initial polymer aggregation is marginally influenced by the contacting interface. Nevertheless, interfacial engineering seems to be appropriate for aggregation tuning, if thermal annealing treatments can be applied^33^. As shown for metal oxides, it can also help to reduce the hydrophilic character^34^, which limits charge separation due to repelling forces pushing donor and acceptor apart from each other. The good agreement between our results based on an H-aggregate analysis of PL spectra and other studies on similar architectures outline that PL measurements are a reliable tool to characterize polymer aggregation, especially for varying film thicknesses.

## Conclusion

In summary, we have shown that the applicability of the H-aggregate model on UV/Vis measurements is limited and can easily be misleading. By comparing experimental P3HT absorbance measurements to calculated spectra we show that the exciton bandwidth determination can evoke the erroneous impression of being thickness dependent. In contrast to ellipsometry measurements, where intrinsic polymer extinction is exclusively measured, UV/Vis spectra are sensitive to reflection at boundary layers or cavity modes. Varying reflection behavior between samples can therefore influence absorbance spectra in a way that a quantitative analysis is not possible, if film thickness or the effective index of refraction is changing between polymer films. We could demonstrate, that reflection always impacts absorbance spectra in such a way that not only transitions relevant for an H-aggregate analysis are affected but also the ratio of aggregated to non-aggregated polymer domains cannot be analyzed quantitatively. For this purpose we suggest to perform more sophisticated measurements such as spectroscopic ellipsometry. Also photoluminescence measurements allow a successful H-aggregate analysis, which is only marginally affected by reflectance and represent a reliable and powerful tool to characterize polymer thin-films. In this regard, we could apply the H-aggregate model on PL spectra to evaluate influences of chelation, surface roughness and hydrophobic/hydrophilic surface characteristics on polymer aggregation.

## Methods

UV/Vis measurements have been performed with an Agilent Cary 5000 UV-Vis-NIR spectrometer equipped with an integrating sphere. For PL measurements a Horiba Fluorolog-3 FL3-122 was used with a Xenon 450 W excitation source. The excitation, as well as the emission light path consist of a double-grating monochromator. A Hamamatsu photomultiplier tube R928P is installed as a detector. PL spectra are measured under an angle of 25° measured to the substrate normal under an excitation wavelength of 530 nm. AFM measurements have been performed using a Bruker Mulitmode 6 in tapping mode.

For all samples under investigation, polished PGO borosilicate glass (D263T) is used. 12 × 22 mm^2^ substrates were cleaned successively in acetone and isopropanol for 10 min in an ultrasonic bath prior to oxygen plasma cleaning for 7 min. Flat 40 nm TiO_2_ films where sputtered, whereas mesoporous films have been fabricated using a Dyesole 18NR-T paste diluted 1:5 g/ml in ethanol. Both flat and mesoporous films where annealed at 450 °C for 60 min using a linear ramp of 7 °C/min. A self-assembled monolayer of [6, 6]-Phenyl C_61_ butyric acid (PCBA) molecules on TiO_2_ has been deposited using a saturated PCBA-bath solution in chlorobenzene (CB). The same procedure holds for decylphosphonic acid (DPA) in a saturated acetonitrile solution. ZnO films were prepared with a 0.5 M sol-gel solution, by dissolving 1.098 g of zinc acetate [Zn(CH_3_COO)_2_.2H_2_O] (Sigma Aldrich) in 10 ml 2-methoxyethanol (Sigma Aldrich), which contains 0.33 ml ethanolamine (Aldrich) as a stabilizer. The ZnO sol-gel solution was spin-casted at 2000 rpm for 40 sec under ambient conditions. After spin-casting, the substrates were annealed at 250 °C for 10 min on a hotplate under ambient conditions. The thin Sb_2_S_3_ layer is prepared by chemical bath deposition (CBD) according to a previously reported procedure with minor adjustments^35^,^36^. In particular, solutions of Na_2_S_2_O_3_ (4 g) in deionized water (25 ml) and SbCl_3_ (650 mg) in acetone (2.5 ml) were prepared and cooled in an ice bath for 90 min. Precursors were mixed into 100 ml of likewise cooled deionized water and samples are immediately put into the mixed solution for 85 min. After chemical bath deposition, the thin Sb_2_S_3_ layers were rinsed with deionized water and promptly dried in a nitrogen stream. Subsequently, the coated backside of the samples was cleaned with hydrochloric acid, and the samples were annealed at 300 °C for 35 min in a nitrogen atmosphere. For perovskite films PbI_2_, PbCl_2_ and MAI with a mole ratio of 1:1:4 are dissolved in DMF using a concentration of 600 mg/ml. The perovskite precursor solution is spin-cast at 3000 rpm in a glovebox and afterwards transferred to a vacuum chamber where it is annealed at 90 °C for 1 min, followed by another annealing step at atmospheric pressure at 100 °C for 10 min. Poly(3-hexylthiophene) (P3HT) is purchased from Rieke Metals with a molecular weight of M_W_ = 90 kDa. The film thickness is adjusted by varying the polymer concentration in CB in a limited range from 5 to 20 mg/ml, while using a spin-speed of 2000 rpm for 120 s The solution is heated to 70 °C for 15 min prior spin-casting, in order to prevent polymer pre-aggregation and gelation in solution^37^.

## Additional Information

**How to cite this article**: Ehrenreich, P. *et al.* H-aggregate analysis of P3HT thin films - Capability and limitation of photoluminescence and UV/Vis spectroscopy. *Sci. Rep.* **6**, 32434; doi: 10.1038/srep32434 (2016).

## Supplementary Material

Supplementary Information
