# Supplementary material for: H-aggregate analysis of P3HT thin films-Capability and limitation of photoluminescence and UV/Vis spectroscopy
Source: Sci Rep. 2016 Sep 1;6:32434. doi: 10.1038/srep32434 (PMC5007523; doi:10.1038/srep32434)
Supplement: Supplementary Information [file srep32434-s1.pdf]

Supplementary Information

H-aggregate analysis of P3HT thin films - Capability and limitation of photoluminescence and UV/Vis spectroscopy

Philipp Ehrenreich, Susanne Birkhold, Eugen Zimmermann, Hao Hu, Kwang-Dae Kim, Jonas Weickert, Thomas Pfadler and Lukas Schmidt-Mende

Department of Physics, University of Konstanz, POB 680, 78467 Konstanz, Germany

UV/Vis results

As it has been discussed in the manuscript, there are significant differences in the absorbance spectra, when comparing a measurement in transmission (Figure S1 top) to measurements obtained in an integrating sphere (Figure S1 bottom). While in transmission measurements the 0-0 transition is successively decreasing with increasing film thickness, there is almost no difference for the measurements in the integrating sphere and especially no systematic behavior. In contrast, the amorphous shoulder is increasing for both cases, with increasing thickness. The reason for this can be found in a change of reflection as demonstrated in the following by calculations.

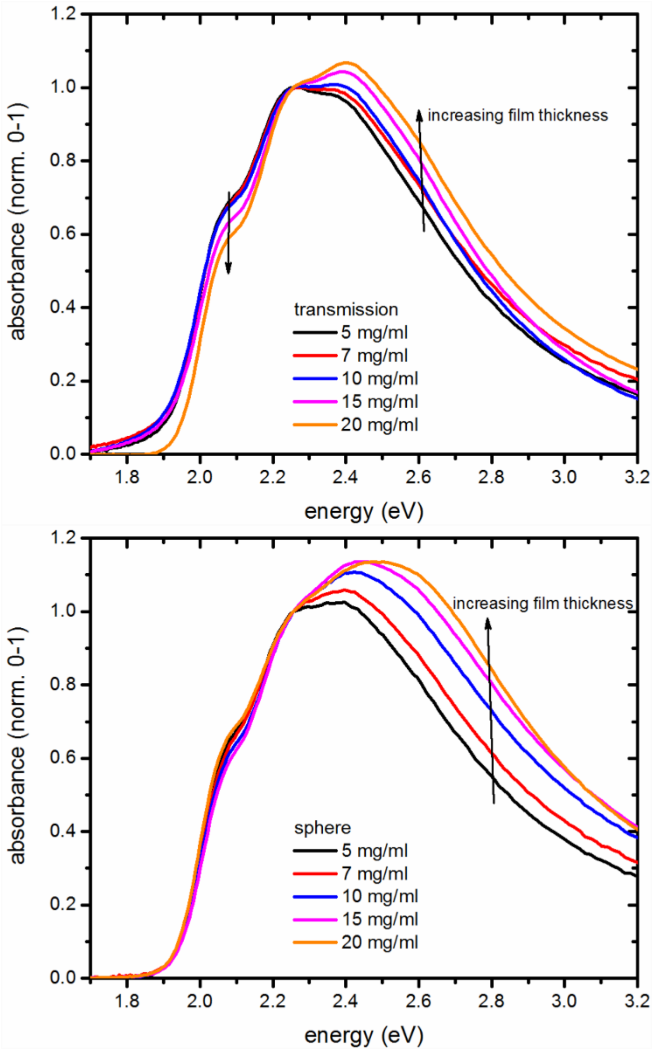

Figure S1: Absorbance measurements in transmission (top) and in reflection - integrating sphere - (bottom) on P3HT films deposited on borosilicate glass.

Transfer matrix calculations

Calculations of absorbance and reflectance spectra were performed using a Matlab script published by Burkhard et al.[1]. To calculate the field profile, coherent interference in the layers and at their interfaces is calculated for each incident wavelength and for specific layer thicknesses. The refractive index of P3HT used for these simulation was determined by ellipsometry for one P3HT film thickness. An infinite layer of glass was assumed as substrate.

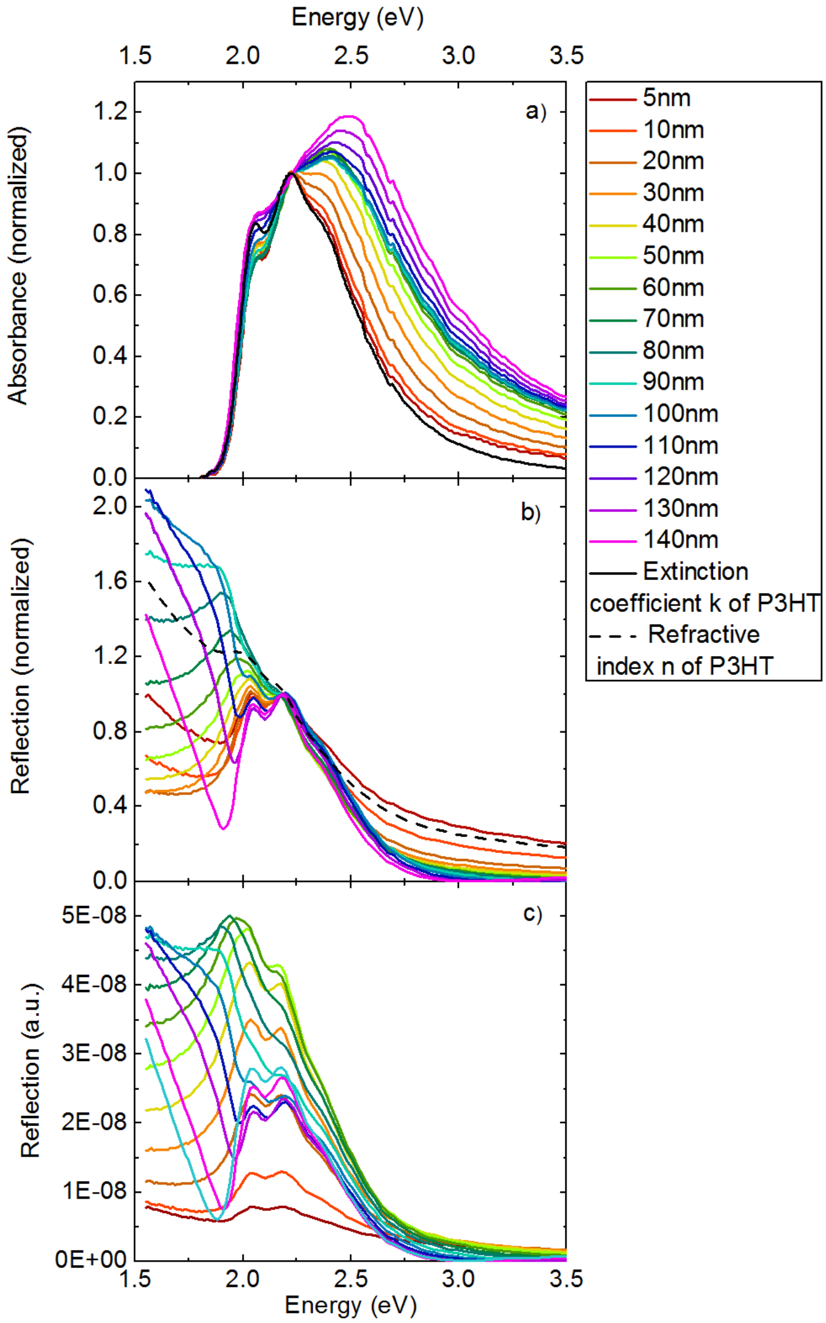

Figure S2: a) Simulated absorbance spectra, determined with the transfer matrix algorithm published by Burkard et al.[1]; the solid black curve shows the extinction coefficient that has been determined by ellipsometry; b) corresponding normalized reflection; the dotted black curve shows the index of refraction that has been determined by ellipsometry; c) total reflection.

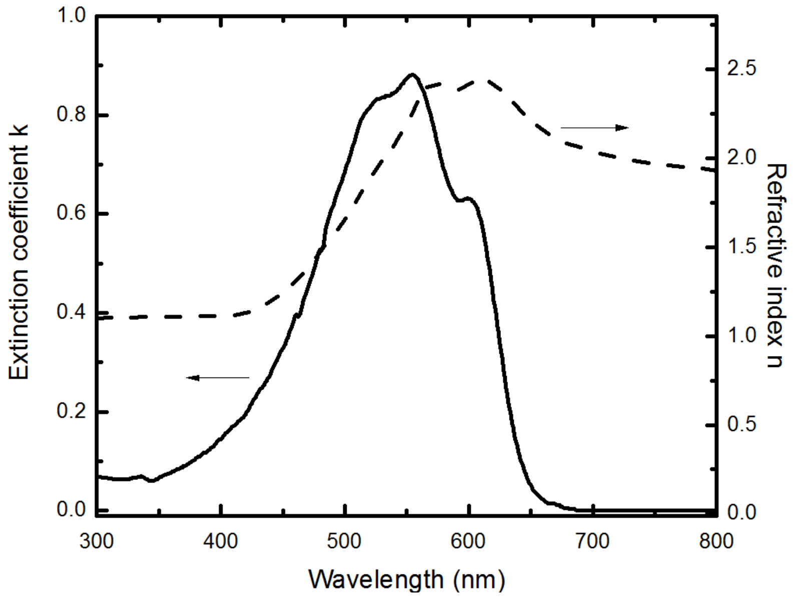

Figure S3: Extinction coefficient k and refractive index n of P3HT used for the calculated absorbance and reflection spectra. The data was measured with a VASE ellipsometer (Woollam).

AFM measurements

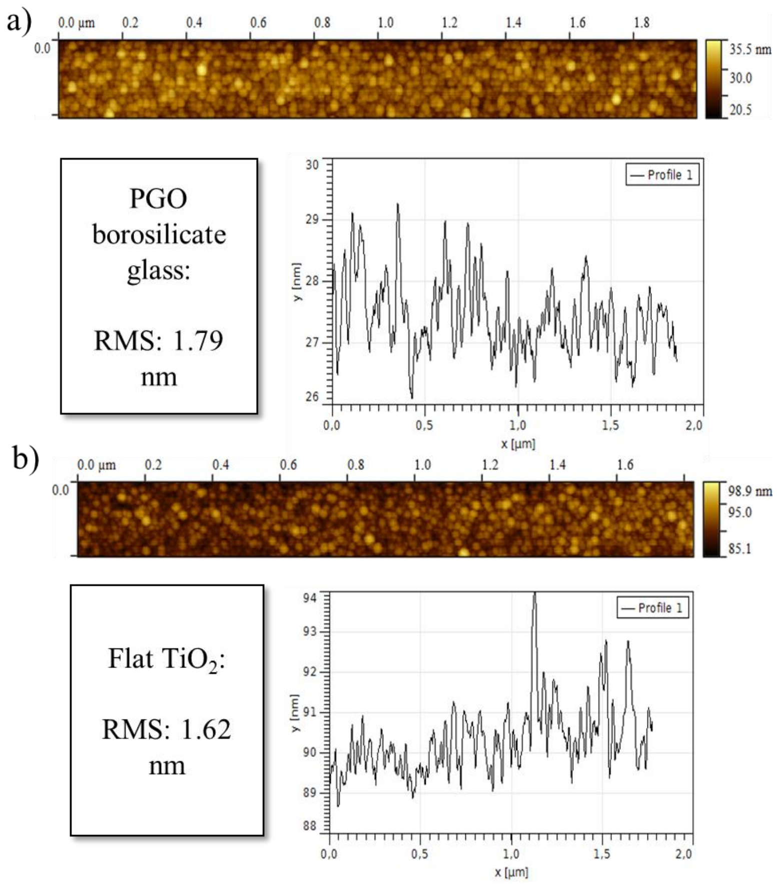

Figure S4: AFM measurement of a borosilicate glass substrate a) and a 40 nm flat TiO_2 layer b) in order to determine the root mean square value of the surface roughness R.

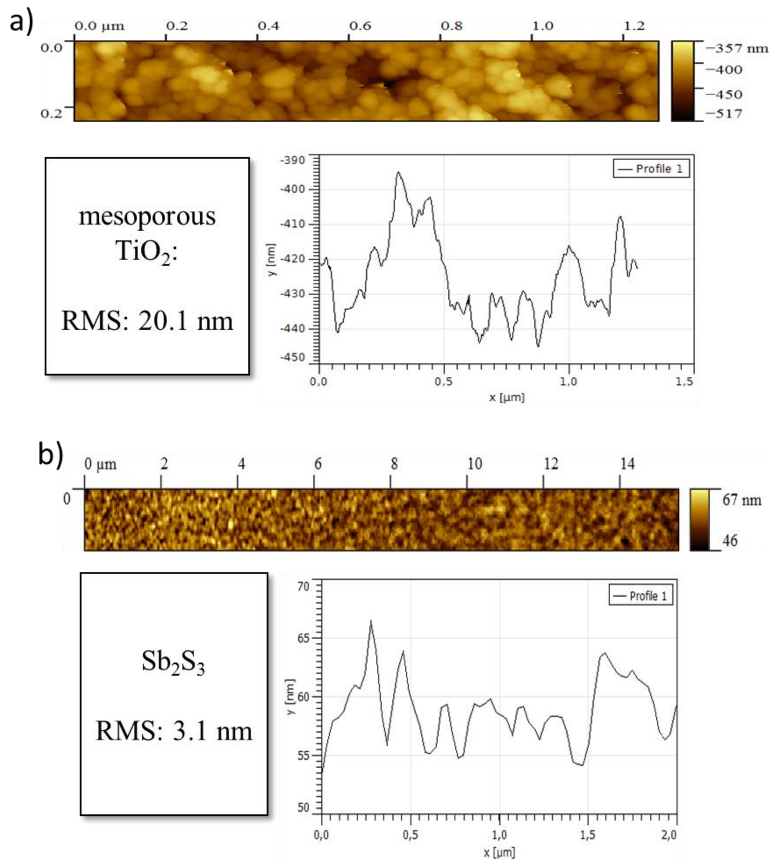

Figure S3: AFM measurement of a mesoporous TiO_2 a) and a Sb_2S_3 substrate b) in order to determine the root mean square value of the surface roughness R.

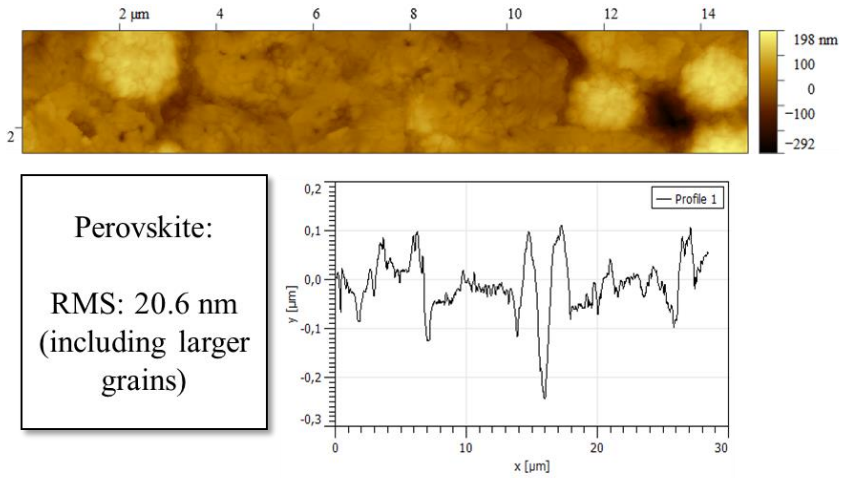

Figure S6: AFM measurement of a perovskite film in order to determine the root mean square value of the surface roughness R; large grains can increase the RMS value significantly, if taken into account.

Reflection losses in photoluminescence spectra

In order to determine the dependence of the refractive index variation at the film/air interface on the measured photoluminescence spectra we have calculated the efficiency of light out-coupling, using Fresnels formula. For this purpose we use a flat air-P3HT interface and the spectrally dependent refractive index of the polymer (displayed in Figure S3) as illustrated in

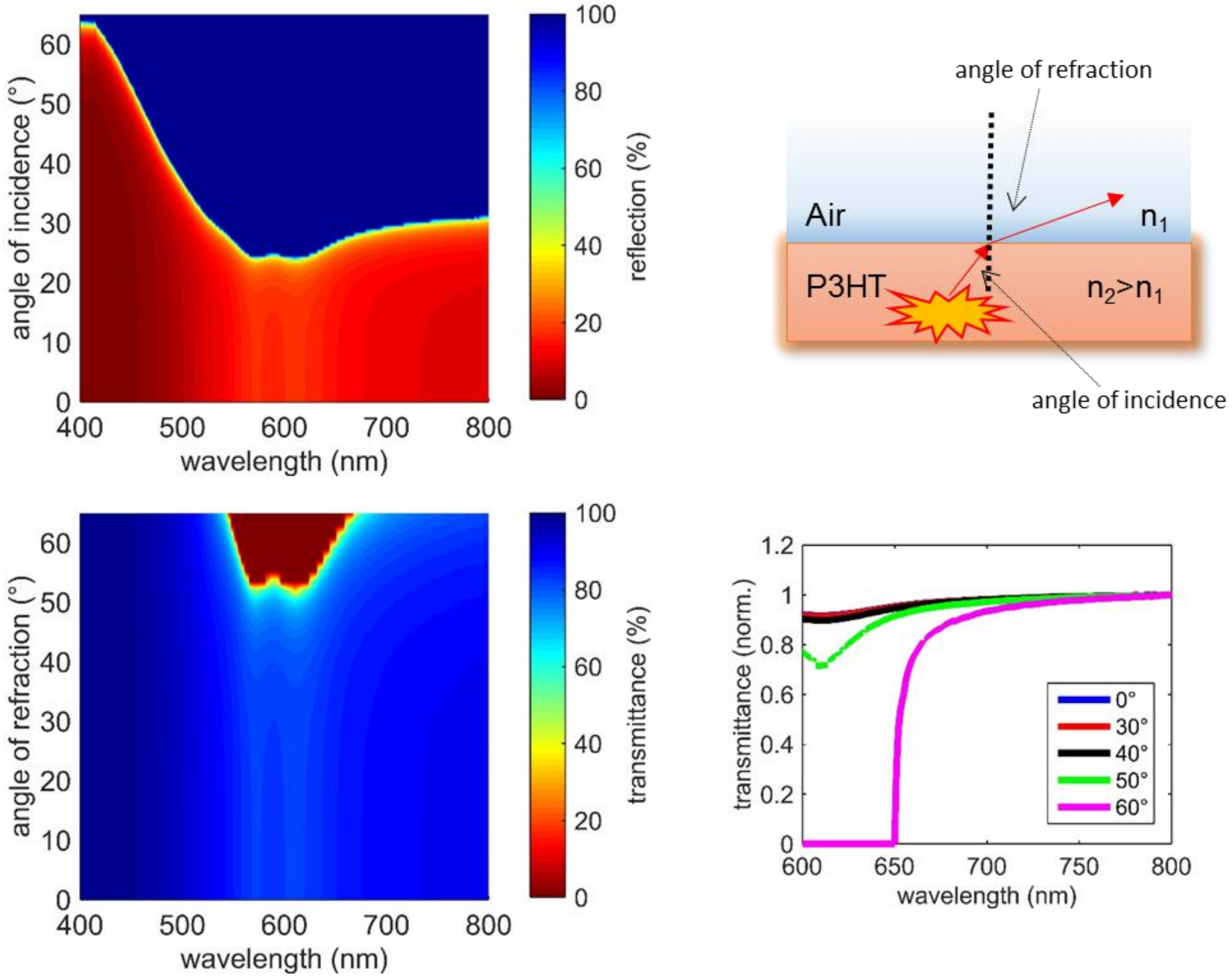

Figure S7. Calculations of light out-coupling at the polymer/air interface using the geometry shown in the top right corner by using the refractive index n of P3HT; Top left: portion of light that is reflected for a specific wavelength depending on the angle of incidence; left bottom: portion of light that is transmitted/out-coupled at the polymer/air interface for a specific wavelength, depending on the angle of refraction; right bottom: efficiency of transmitted/out-coupled light as a function of wavelength for different angles of refraction.

Figure S7 on the top right. Since the polymer luminescence is isotropic in all direction, only a fraction of light can be coupled out, i.e. an angle of incidence that is well below the angle of total reflection. Photoluminescence for P3HT occurs well above 620 nm. Therefore light out-coupling works efficient until the solid angle of approximately $\pi/9$- $\pi/6$. For larger angles of incidence, emission either occurs towards the other half-space direction or is totally reflected towards the air interface. More important for the PL spectrum is the fraction of transmitted light for a given angle of detection (angle of refraction). Almost all light is transmitted out of the film and spectral differences are small till 50°. If the PL spectrum is measured under a larger angle closer to the angle of total reflection, stronger influences of spectrally dependent inefficient light out-coupling get more relevant. For angles apart from this limit, the spectral

out-coupling efficiency is not varying significantly (Figure S7 bottom right). Normalizing the out-coupling efficiency to 800 nm, one detects approximately 8-10 % less photons at 620 nm. Between the 0-0 transition and the 0-1 transition the difference is smaller than 5 %. This effect is neglected in our PL analysis, since it is comparably small and the contribution is the same for all spectra, independent of film thickness.

Photoluminescence measurements

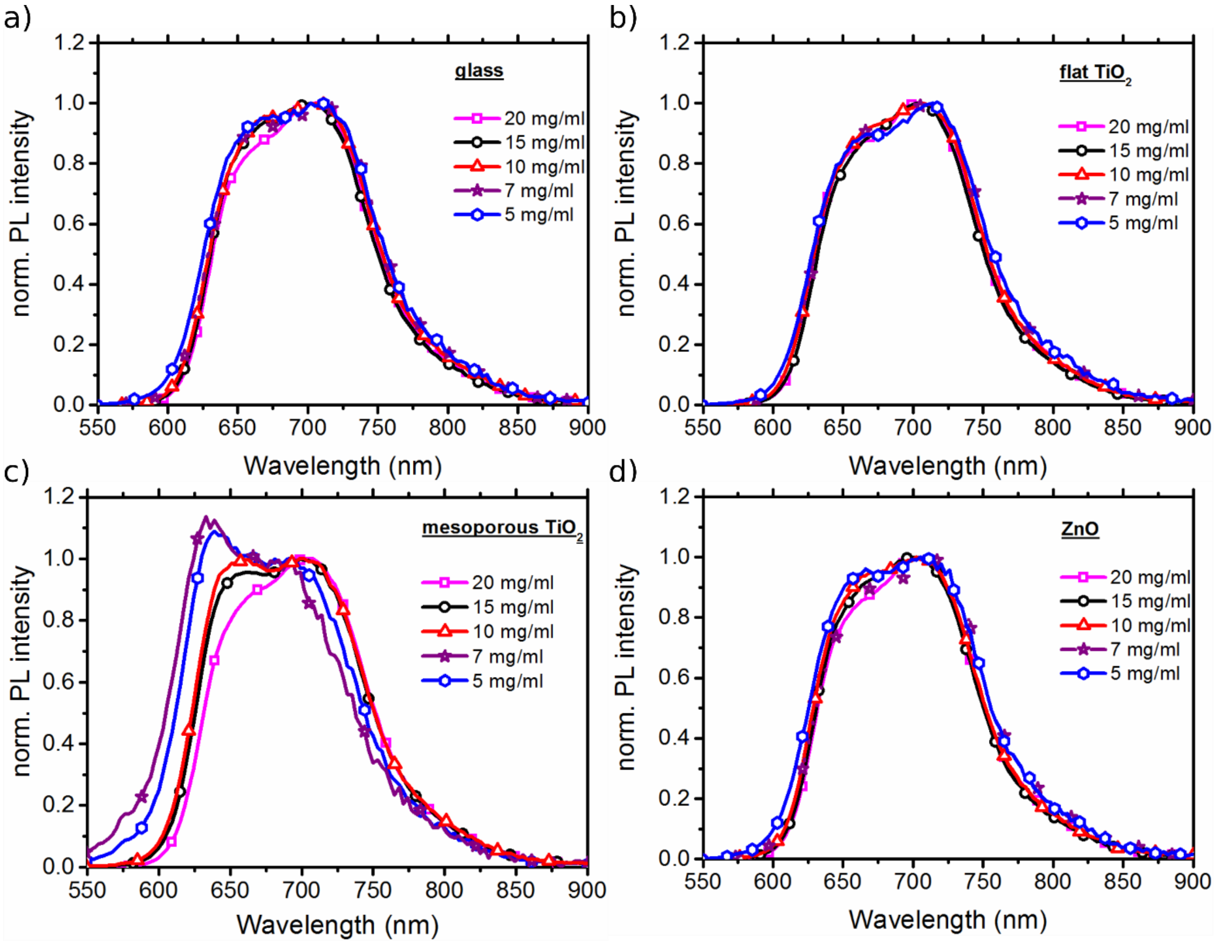

Figure S8: Normalized photoluminescence (PL) spectra for varying film thicknesses resulting after spin-casting different solution concentrations of P3HT (5-20 mg/ml) on a) borosilicate glass b) a flat 40 nm TiO_2 film, c) a mesoporous TiO_2 film and d) a 40 nm flat ZnO film.

Figures S8 and S9 summarize photoluminescence spectra collected of P3HT films spin-cast on different substrates using varying polymer concentrations in solution. The photoluminescence spectra are normalized to the 0-1 transition, that is insensitive to the nature of aggregation[2]. The absolute film thickness cannot be compared, due to different wetting properties. For this reason, quantitative comparisons shall not be made. Based on the AFM measurements presented above, it is obvious that the spectra are significantly influenced by the surface roughness for thinner films (lighter concentrations). This can be seen in an increase of the 0-0 transition, a slight blue-shift and a broadening of the spectra for thinner films on mesoporous TiO_2 and Sb_2S_3 substrates.

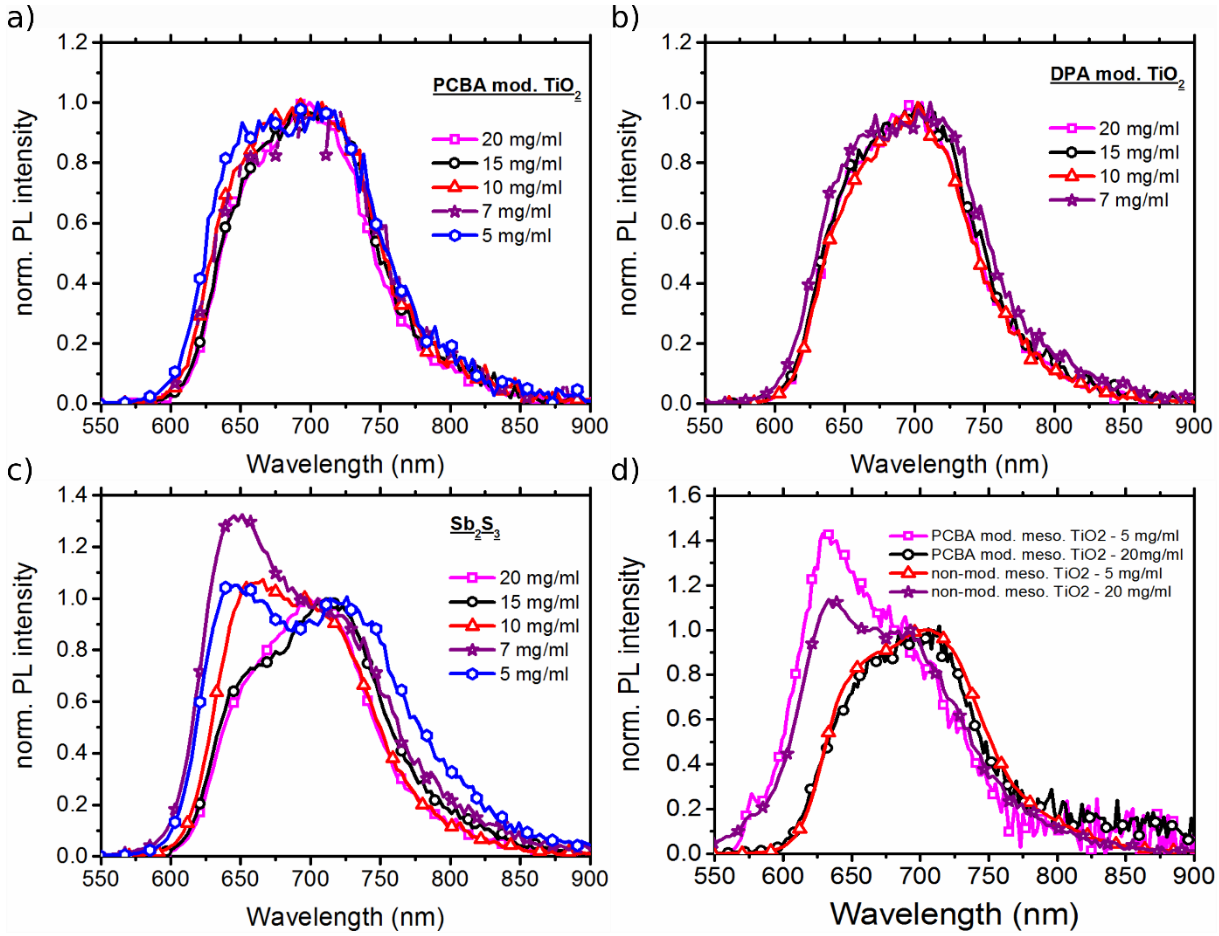

Figure S9: Normalized photoluminescence (PL) spectra for varying film thicknesses resulting after spin-casting different solution concentrations of P3HT (5-20 mg/ml) on a) a self-assembled monolayer (SAM) PCBA on flat TiO_2 b) a SAM DPA on flat TiO_2 and a Sb_2S_3 film c). In d) a SAM PCBA on mesoporous TiO_2 is compared to a pristine mesoporous TiO_2 surface.

References

1. G. F. Burkhard, E. T. Hoke and M. D. McGehee, *Advanced Materials*, 2010, **22**, 3293-3297.
2. F. C. Spano and C. Silva, *Annual Review of Physical Chemistry*, 2014, **65**.
